# Supplementary figures and images for: Sleep quality and the evolution of the COVID-19 pandemic in five European countries
Source: PLoS One. 2022 Dec 28;17(12):e0278971. doi: 10.1371/journal.pone.0278971 (PMC9797060; doi:10.1371/journal.pone.0278971)

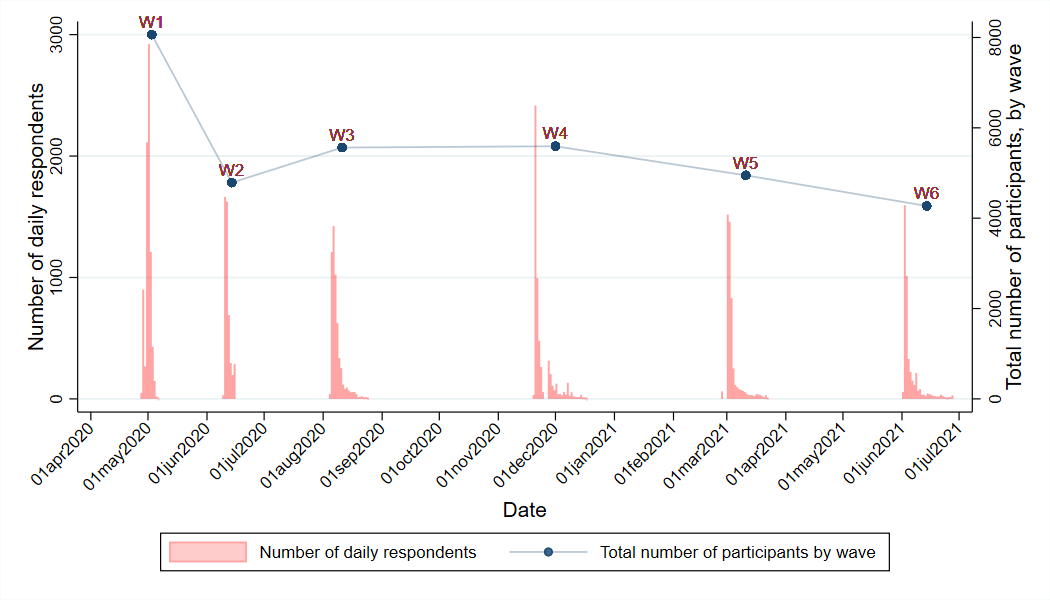

Supplement: S1 Fig — Notes: The figure refers to all survey respondents in COME-HERE. Each histogram bar represents the number of respondents in a given day (y-axis on the left). Blue dots indicate the number of total respondents per wave (y-axis on the right). (TIF) [file pone.0278971.s001.tif]
