# Supplementary material for: Sleep quality and the evolution of the COVID-19 pandemic in five European countries
Source: PLoS One. 2022 Dec 28;17(12):e0278971. doi: 10.1371/journal.pone.0278971 (PMC9797060; doi:10.1371/journal.pone.0278971)
Supplement: S1 Table — Notes: These are linear regressions. The sample here is respondents coming from the four 2020 waves and the first two 2021 waves of the COME-HERE survey. All the continuous variables are standardised over the estimation sample. Standard errors in parentheses are clustered at the individual level. All regressions include wave and country fixed-effects *, **, and *** respectively indicate significance levels of 10%, 5% and 1%. (DOCX) [file pone.0278971.s002.docx]

|  | Sleep Quality (1-7 – standardised) | | | |
| --- | --- | --- | --- | --- |
|  | (1) | (2) | (3) | (4) |
| Average Daily Deaths/100,000 inhabitants | -0.026^***^ | -0.036^***^ | -0.035^***^ | -0.033^***^ |
| (4-week average) | (0.010) | (0.010) | (0.008) | (0.010) |
|  |  |  |  |  |
| Age: 30 to 39 years |  | -0.085^**^ |  |  |
|  |  | (0.037) |  |  |
|  |  |  |  |  |
| Age: 40 to 49 years |  | -0.074^**^ |  |  |
|  |  | (0.037) |  |  |
|  |  |  |  |  |
| Age: 50 to 59 years |  | 0.007 |  |  |
|  |  | (0.038) |  |  |
|  |  |  |  |  |
| Age: 60 to 69 years |  | 0.283^***^ |  |  |
|  |  | (0.038) |  |  |
|  |  |  |  |  |
| Age: 70+ years |  | 0.384^***^ |  |  |
|  |  | (0.048) |  |  |
|  |  |  |  |  |
| Female |  | -0.139^***^ |  |  |
|  |  | (0.022) |  |  |
|  |  |  |  |  |
| Education (ref: Lower-Secondary) |  |  |  |  |
| Upper-Secondary Education |  | 0.006 |  |  |
|  |  | (0.031) |  |  |
|  |  |  |  |  |
| Post-Secondary Education |  | 0.006 |  |  |
|  |  | (0.032) |  |  |
|  |  |  |  |  |
| Living with a Partner |  | 0.064^***^ |  |  |
|  |  | (0.024) |  |  |
|  |  |  |  |  |
| Children at Home |  | -0.023 |  |  |
|  |  | (0.027) |  |  |
|  |  |  |  |  |
| Population Density (ref: isolated dwelling) |  |  |  |  |
| Less than 2,000 |  | -0.085^**^ |  |  |
|  |  | (0.037) |  |  |
|  |  |  |  |  |
| Between 2,000 and 10,000 |  | -0.074^**^ |  |  |
|  |  | (0.037) |  |  |
|  |  |  |  |  |
| Between 10,000 and 50,000 |  | 0.007 |  |  |
|  |  | (0.038) |  |  |
|  |  |  |  |  |
| Between 50,000 and 100,000 |  | 0.283^***^ |  |  |
|  |  | (0.038) |  |  |
|  |  |  |  |  |
| More than 100,000 |  | 0.384^***^ |  |  |
|  |  | (0.048) |  |  |
|  |  |  |  |  |
| Log of Household Equivalised Income (in |  | 0.086^***^ | 0.019 | 0.019 |
| PPP) |  | (0.011) | (0.012) | (0.012) |
|  |  |  |  |  |
| Employed |  | 0.019 | 0.022 | 0.023 |
|  |  | (0.028) | (0.033) | (0.033) |
|  |  |  |  |  |
| Stringency Index (2-week average) |  |  |  | -0.007 |
|  |  |  |  | (0.008) |
|  |  |  |  |  |
| Economic Support Index (2-week average) |  |  |  | -0.008 |
|  |  |  |  | (0.008) |
| Observations | 27728 | 27728 | 27728 | 27728 |
