# Supplementary material for: Sleep quality and the evolution of the COVID-19 pandemic in five European countries
Source: PLoS One. 2022 Dec 28;17(12):e0278971. doi: 10.1371/journal.pone.0278971 (PMC9797060; doi:10.1371/journal.pone.0278971)
Supplement: S2 Table — Notes: The sample here is respondents coming from the four 2020 waves and the first two 2021 waves of the COME-HERE survey. The dependent variable is the continuous sleep quality, except in column (3) where the dependent variable is a dummy equal to one for high sleep quality (sleep quality above the median). The continuous sleep quality and the average daily deaths variables are standardised over the estimation sample. Standard errors in parentheses are clustered at the individual level, except in column (5) where they are clustered at the four-week average daily deaths per 100,000 inhabitants’ level. These are linear regressions, except in column (2) where we used the BUC estimator [60]. Longitudinal and cross-sectional weights were respectively used in columns (6) and (7). The individual controls are age categories, gender, education, parenthood, relationship status, and population density (all measured at Wave 1), the log of equivalent household disposable income in PPP, and a dummy for the employment status. The pandemic policies are the two-week averages of the Stringency Index and Economic Support Index. All regressions include wave and country fixed-effects *, **, and *** respectively indicate significance levels of 10%, 5% and 1%. (DOCX) [file pone.0278971.s003.docx]

|  | Sleep Quality | | | | | | |
| --- | --- | --- | --- | --- | --- | --- | --- |
|  | (1) | (2) | (3) | (4) | (5) | (6) | (7) |
| Average Daily Deaths/100,000 | -0.033^***^ | -0.101^***^ | -0.022^***^ |  | -0.033^***^ | -0.031^***^ | -0.039^***^ |
| inhabitants (4-week average) | (0.010) | (0.031) | (0.005) |  | (0.009) | (0.011) | (0.012) |
|  |  |  |  |  |  |  |  |
| Average Daily Deaths/100,000 |  |  |  | -0.020^*^ |  |  |  |
| inhabitants (2-week average) |  |  |  | (0.011) |  |  |  |
| Observations | 27728 | 27728 | 27728 | 27728 | 27728 | 27728 | 27728 |
