# Supplementary material for: Sleep quality and the evolution of the COVID-19 pandemic in five European countries
Source: PLoS One. 2022 Dec 28;17(12):e0278971. doi: 10.1371/journal.pone.0278971 (PMC9797060; doi:10.1371/journal.pone.0278971)
Supplement: S3 Table — Notes: These are linear regressions. The sample here is respondents coming from the four 2020 waves and the first two 2021 waves of the COME-HERE survey. Sleep quality and the average daily deaths variable are standardised over the estimation sample. Standard errors in parentheses are clustered at the individual level. The individual controls are age categories, gender, education, parenthood, relationship status, and population density (all measured at Wave 1), the log of equivalent household disposable income in PPP, and a dummy for the employment status. The pandemic policies are the two-week averages of the Stringency Index and Economic Support Index. All regressions include wave and country fixed-effects *, **, and *** respectively indicate significance levels of 10%, 5% and 1%. (DOCX) [file pone.0278971.s004.docx]

|  | Sleep Quality (1-7 – standardised) | | | | |
| --- | --- | --- | --- | --- | --- |
|  | (1) | (2) | (3) | (4) | (5) |
| Average Daily Deaths/100,000 inhabitants | -0.021^**^ | -0.022^**^ | -0.032^***^ | -0.020^**^ | -0.029^**^ |
| (4-week average) | (0.010) | (0.010) | (0.010) | (0.010) | (0.011) |
|  |  |  |  |  |  |
| Interacted with: |  |  |  |  |  |
| Female | -0.025^***^ |  |  |  |  |
|  | (0.009) |  |  |  |  |
|  |  |  |  |  |  |
| Young |  | -0.021^**^ |  |  |  |
|  |  | (0.009) |  |  |  |
|  |  |  |  |  |  |
| Lower-Secondary Education |  |  | -0.001 |  |  |
|  |  |  | (0.011) |  |  |
|  |  |  |  |  |  |
| Children in the Household |  |  |  | -0.036^***^ |  |
|  |  |  |  | (0.009) |  |
|  |  |  |  |  |  |
| Low Household Income |  |  |  |  | -0.006 |
|  |  |  |  |  | (0.009) |
| Observations | 27728 | 27728 | 27728 | 27728 | 27728 |
