# Supplementary material for: Sleep quality and the evolution of the COVID-19 pandemic in five European countries
Source: PLoS One. 2022 Dec 28;17(12):e0278971. doi: 10.1371/journal.pone.0278971 (PMC9797060; doi:10.1371/journal.pone.0278971)
Supplement: Attachment — Submitted filename: Response to Reviewer.docx [file pone.0278971.s006.docx]

**Referee 1**

Thank you very much for your careful reading and comments on this paper. We found your comments very helpful and we have revised the paper accordingly. Our detailed responses to these comments appear below.

**Major Comments:**

1. **I have only few concerns. Recent literature often considers the size of a municipality (or population density of the municipality of residence) as an important control in order to split urban areas and rural ones.**

**My suggestion is to control for this covariate.**

Thank you for pointing this out, population density can indeed be an important covariate to consider in our context. In the COME-HERE dataset, the following question is collected in wave 1:

Q17 What type of area/city/town/village do you live in?

-	population of more than 100 000 (1)

-	population between 100 000 and 50 000 (2)

-	population between 50 000 and 10 000 (3)

-	population between 10 000 and 2 000 (4)

-	population less than 2 000 (5)

-	isolated dwelling (6)

We now include this categorical variable in the list of ‘individual controls’ used in Tables 2, 3, and A1 (Table A1, displaying the full list of coefficients for our main results, is reported below for convenience). We additionally amended the descriptive statistics, table footnotes and manuscript to reflect the introduction of this new control.

As revealed by the second column of Table A1 below, population density does correlate significantly with sleep quality. However, the estimate attracted by the average daily COVID-19 deaths remained the same. We can then arguably consider population density to be a relevant control but not an omitted variable.

Table A1: Sleep Quality and COVID-19 Deaths – Full Pooled and Panel Results

|  | Sleep Quality (1-7 – standardised) | | | |
| --- | --- | --- | --- | --- |
|  | (1) | (2) | (3) | (4) |
| Average Daily Deaths/100,000 inhabitants | -0.026^***^ | -0.036^***^ | -0.035^***^ | -0.033^***^ |
| (4-week average) | (0.010) | (0.010) | (0.008) | (0.010) |
|  |  |  |  |  |
| Age: 30 to 39 years |  | -0.085^**^ |  |  |
|  |  | (0.037) |  |  |
|  |  |  |  |  |
| Age: 40 to 49 years |  | -0.074^**^ |  |  |
|  |  | (0.037) |  |  |
|  |  |  |  |  |
| Age: 50 to 59 years |  | 0.007 |  |  |
|  |  | (0.038) |  |  |
|  |  |  |  |  |
| Age: 60 to 69 years |  | 0.283^***^ |  |  |
|  |  | (0.038) |  |  |
|  |  |  |  |  |
| Age: 70+ years |  | 0.384^***^ |  |  |
|  |  | (0.048) |  |  |
|  |  |  |  |  |
| Female |  | -0.139^***^ |  |  |
|  |  | (0.022) |  |  |
|  |  |  |  |  |
| Education (ref: Lower-Secondary) |  |  |  |  |
| Upper-Secondary Education |  | 0.006 |  |  |
|  |  | (0.031) |  |  |
|  |  |  |  |  |
| Post-Secondary Education |  | 0.006 |  |  |
|  |  | (0.032) |  |  |
|  |  |  |  |  |
| Living with a Partner |  | 0.064^***^ |  |  |
|  |  | (0.024) |  |  |
|  |  |  |  |  |
| Children at Home |  | -0.023 |  |  |
|  |  | (0.027) |  |  |
|  |  |  |  |  |
| Population Density (ref: isolated dwelling) |  |  |  |  |
| Less than 2,000 |  | -0.085^**^ |  |  |
|  |  | (0.037) |  |  |
|  |  |  |  |  |
| Between 2,000 and 10,000 |  | -0.074^**^ |  |  |
|  |  | (0.037) |  |  |
|  |  |  |  |  |
| Between 10,000 and 50,000 |  | 0.007 |  |  |
|  |  | (0.038) |  |  |
|  |  |  |  |  |
| Between 50,000 and 100,000 |  | 0.283^***^ |  |  |
|  |  | (0.038) |  |  |
|  |  |  |  |  |
| More than 100,000 |  | 0.384^***^ |  |  |
|  |  | (0.048) |  |  |
|  |  |  |  |  |
| Log of Household Equivalised Income (in |  | 0.086^***^ | 0.019 | 0.019 |
| PPP) |  | (0.011) | (0.012) | (0.012) |
|  |  |  |  |  |
| Employed |  | 0.019 | 0.022 | 0.023 |
|  |  | (0.028) | (0.033) | (0.033) |
|  |  |  |  |  |
| Stringency Index (2-week average) |  |  |  | -0.007 |
|  |  |  |  | (0.008) |
|  |  |  |  |  |
| Economic Support Index (2-week average) |  |  |  | -0.008 |
|  |  |  |  | (0.008) |
| Observations | 27728 | 27728 | 27728 | 27728 |

*Notes*: These are linear regressions. The sample here is respondents coming from the four 2020 waves and the first two 2021 waves of the COME-HERE survey. All the continuous variables are standardised over the estimation sample. Standard errors in parentheses are clustered at the individual level. All regressions include wave and country fixed-effects ^*^, ^**^, and ^***^ respectively indicate significance levels of 10%, 5% and 1%.

As the question on population density was only asked once, coefficients for this control are no longer estimated whenever we introduce individual fixed-effects. We do not however worry from potential confounding coming from movers, as mobility was substantially reduced during the Covid-19 pandemic. People who did move, did so mostly from high-density to low-density areas and thus might contribute to an attenuation, rather than an inflation, of our results (people moving out of densely-populated areas are arguably less exposed to worry and anxiety linked to Covid-19).

2. **In addition, I don't see the estimates for the respondents' education level for every level, but I can see also a dichotomization (low level vs. post-secondary) Is it correct? I would like to see more stratification and an homogeneity in the label of the education categories.**

Thank you, we did indeed make a dichotomisation of education (post-secondary vs lower). We do however have access to a finer detail when it comes to education. While the education systems of the five countries in our sample have some differences, we can always distinguish between lower-secondary and upper-secondary education in all countries. We now updated our definition of education and use it as a categorical variable that has three levels (lower-, upper-, and post-secondary) instead of two. The manuscript and tables now reflect this change and descriptive statistics in Table 1 have been updated accordingly. Table A1, reported above in this document, contains the coefficients attached to the new education categories.

As you correctly spotted, we had a “low education” category in Table A3 that we did not define anywhere. In the context of our newly defined education variable, this would be category 1, “lower-secondary education”. The label has now been changed to reflect the new categorisation.
